# Alleviation of arthritis through prevention of neutrophil extracellular traps by an orally available inhibitor of protein arginine deiminase 4

**DOI:** 10.1038/s41598-023-30246-2

**Published:** 2023-02-23

**Authors:** Chandru Gajendran, Shoichi Fukui, Naveen M. Sadhu, Mohammed Zainuddin, Sridharan Rajagopal, Ramachandraiah Gosu, Sarah Gutch, Saeko Fukui, Casey E. Sheehy, Long Chu, Santosh Vishwakarma, D. A. Jeyaraj, Gurulingappa Hallur, Denisa D. Wagner, Dhanalakshmi Sivanandhan

**Affiliations:** 1 Program in Cellular and Molecular Medicine, Boston Children’s Hospital, Boston, MA 02115 USA; 2 Department of Pediatrics, Harvard Medical School, Boston, MA 02115 USA; 3 Division of Hematology/Oncology, Boston Children’s Hospital, Boston, MA 02125 USA; 4 Jubilant Therapeutics Inc., Bedminster, NJ USA; 5 Jubilant Biosys Limited, Bangalore, India

**Keywords:** Rheumatoid arthritis, Autoimmunity, Pharmacodynamics

## Abstract

Protein arginine deiminases (PAD) 4 is an enzyme that catalyzes citrullination of protein and its role in autoimmune diseases has been established through clinical genetics and gene knock out studies in mice. Further, studies with PAD4 – deficient mice have shown that PAD4 deficiency does not lead to increased infection or immune suppression, which makes PAD4 an attractive therapeutic target for auto-immune and inflammatory diseases. PAD4 has critical enzymatic role of promoting chromatin decondensation and neutrophil extracellular traps (NETs) formation that is associated with a number of immune-mediated pathological conditions. Here, we present a non-covalent PAD4 inhibitor JBI-589 with high PAD4 isoform selectivity and delineated its binding mode at 2.88 Å resolution by X-ray crystallography. We confirmed its effectiveness in inhibiting NET formation in vitro. Additionally, by using two mouse arthritis models for human rheumatoid arthritis (RA), the well-known disease associated with PAD4 clinically, we established its efficacy in vivo. These results suggest that JBI-589 would be beneficial for both PAD4 and NET-associated pathological conditions.

## Introduction

Citrullination is a form of protein post-translational modification characterized by the conversion of positively charged peptidyl-arginine to neutral peptidyl-citrulline, thus modifying the charge, epitopes, and function of proteins. Citrullination is catalyzed by the peptidylarginine deiminases (PADs), of which there are five isozymes (PAD1-4, PAD6). Citrullination has essential roles in various physiological processes, including cell differentiation, transcriptional regulation, and immune response^1^.

PAD4 is expressed by eosinophils, neutrophils^2^, monocyte and macrophages^3^, and is the only isozyme with a canonical nuclear localization sequence^4^. PAD4 has multiple effects on cellular processes such as cell proliferation^5^ and cytokine production^6^. In addition, PAD4 plays a key role in releasing neutrophil extracellular traps (NETs) by citrullinating histones, which leads to the decondensation of chromatin^7^. NETs are produced at varying levels and kinetics in response to different stimuli. Based on the data from our lab, all these stimuli lead to citrullination of histones and require PAD4 at least to some extent. PAD4 is implicated in other cellular events linked to NETosis such as NLRP3 inflammasome activation and nuclear rupture^8,9^. Some stimuli such as PMA internally liberate serine proteases and they in turn cleave the citrullinated tails of histones which become no longer detectable by antibodies. In the presence of PMSF (a potent serine protease inhibitor), citrullination is detected in PMA stimulated neutrophils^10^.

Genetic studies have shown the relationship between PAD4 and autoimmune diseases. Rheumatoid arthritis (RA) is the most well-known autoimmune disease connected with *PADI4*, the gene encoding human PAD4 protein. Genome-wide association studies (GWAS) showed the relationship between *PADI4* single nucleotide polymorphism and RA in Asian cohorts^11^, followed by consistent results from populations of European ancestry^12^. GWAS also demonstrated the correlation of *PADI4* polymorphism with IgA nephropathy^13^ as well as systemic lupus erythematosus (SLE)^14^.

The impacts of *Padi4*, encoding mouse PAD4 protein, on diseases have been shown in animal disease models. *Padi4* deficient mice revealed the amelioration of arthritis severity in mouse arthritis models^15–18^. In addition, *Padi4* deficiency has demonstrated improved clinical parameters^19^ and amelioration of organ damage^20^ in the mouse lupus model^1^.

As of today, there is ample evidence to suggest that PAD4 could be a promising target for multiple autoimmune diseases as well as cancer. Several pan-PAD and few PAD4 inhibitors have been demonstrated^4^ to be efficacious in inhibiting neutrophil extracellular traps formation^21^ in murine models of arthritis^22^, psoriasis and multiple myeloma^23^. However, no PAD4 inhibitor has been approved so far for human use and therefore further efforts on developing PAD4 inhibitors with appropriate Absorption, Distribution, Metabolism, and Excretion (ADME) properties, safety and efficacy will be highly valuable for treating multiple human diseases.

Therefore, in an effort to identify selective and orally bioavailable PAD4 inhibitors, we synthesized a series of molecules using rational drug design. Few of the scaffolds were subjected to co-crystallization studies to evaluate the binding modes. One of our lead molecules JBI-589 is highly potent and selective for PAD4 with excellent ADME and pharmacokinetics (PK) properties and demonstrated strong efficacy in mouse arthritis models in vivo.

## Results

### Biochemical and structural studies of PAD4

**Figure 1:**
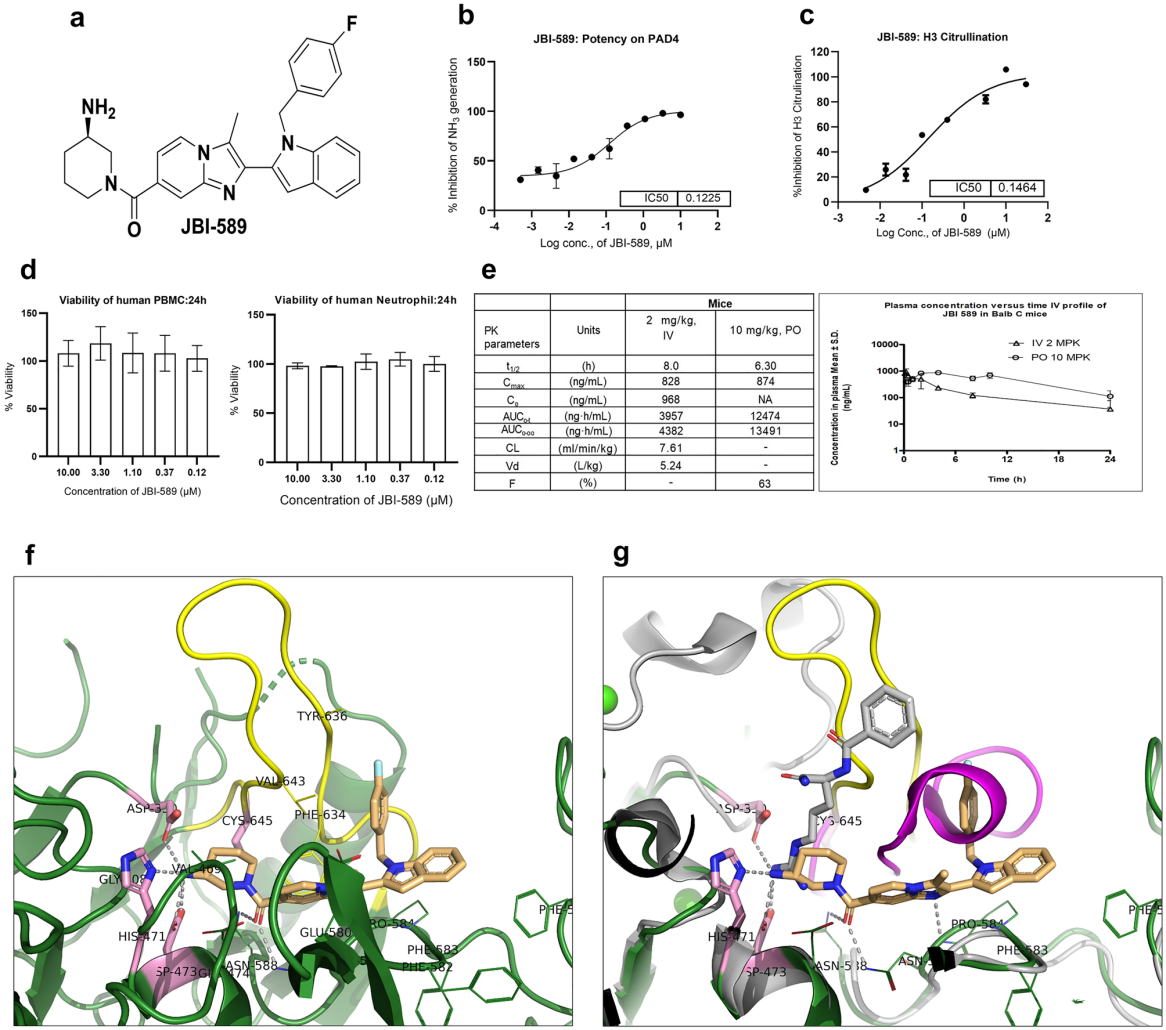
Structure, Biochemical and cellular potency of JBI-589. (**a**). Chemical structure of JBI-589 (**b**) Potency of JBI-589 in PAD4 biochemical assay measuring ammonia release (**c**) Potency of JBI-589 in inhibiting calcium ionophore induced citrullination using purified human neutrophils. (**d**) Effect of JBI-589 on human neutrophil and PBMC viability. (**e**) Oral (10 mg/kg) and intravenous (2 mg/kg) pharmacokinetic parameters of JBI-589 in Balb C mice. (**f**) JBI-589 bound to PAD4 at the active site. JBI-589 is shown in sticks (in light orange color). Catalytic residues (Asp-350, His-471, Asp-473, Cys-645) are shown in pink. Residues within 4 Å of JBI-589 are shown in lines. Inhibitor-protein interactions are shown in grey dotted lines. (**g**) Overlay of JBI-589 bound PAD4 (green, calcium-free) onto BAA bound PAD4 (grey, PDB: 1WDA). JBI-589 (light orange sticks) and substrate-like inhibitor, BAA (grey sticks) are oriented ~ 70° to each other. Region aa630-aa647 shows conformational change and movement from helix (in BAA-PAD4, magenta) to ß-hairpin/loop (in JBI-589-PAD4, yellow). Calcium ions seen in BAA-PAD4 are shown in green spheres. For clarity, few residues are trimmed.

The chemical structure of JBI-589 is shown in Fig. 1a. Synthetic scheme is given in supplementary methods (Suppl. notes). JBI-589 was screened against recombinant human PAD4 enzyme in ammonia release assay at 10 semi-log concentrations where it showed dose-dependent inhibition of PAD4 enzymatic activity with a half-maximum inhibitory concentration (IC_50_) of 0.122 µM (Fig. 1b). In a similar assay format, JBI-589 was screened against human recombinant PAD1, PAD2, PAD3 and PAD6 enzymes at 10 semi-log concentrations where JBI-589 did not have any inhibitory activity against these enzymes even at the highest concentration tested (30 µM) clearly showing that JBI-589 is highly selective for PAD4 (supplementary Fig. S1d). JBI-589 treatment resulted in a dose-dependent inhibition of histone H3 citrullination in human neutrophils induced by 25 µM calcium ionophore, as observed by ELISA. In this assay, JBI-589 showed an EC50 of 0.146 µM (Fig. 1c). Treatment of human neutrophils and PBMCs with JBI-589 for 24 h at various concentrations resulted in minimal or no changes in the viability of neutrophils and PBMCs up to 10 µM concentration (Fig. 1d). From thermal shift assay, binding of JBI-589 to PAD4 was assessed where, in the presence of JBI-589 melting temperature (Tm) of PAD4 increased by 7 °C when compared to apo-PAD4 (Supplementary Fig. S1a and b).

PAD4 forms head-to-tail dimer with each monomer having one catalytic domain at C-terminus preceded by N-terminal IgG subdomains 1 and 2 (Supplementary Fig. S1c). Here the reported structure was obtained in calcium-free form with JBI-589 bound at the catalytic pocket with binding mode distinctly different from the substrate-like inhibitor (for e.g., benzoyl-arginine amide (BAA), PDB: 1WDA reported with mutant PAD4 i.e., Cys645Ala). The binding mode of JBI-589 was found to be nearly perpendicular (~ 70°) to BAA orientation such that 3 of the catalytic residues (Asp-350, His-471, Asp-473) positioned almost in-close proximity for both binding modes whereas 4th catalytic residue, Cys-645 is located ~ 7.5 Å away from JBI-589 in a direction perpendicular to the plane formed by BAA and JBI-589 binding modes but proximal to BAA. Upon JBI-589 binding to PAD4, conformational change and movement is seen for the region aa630–aa647 from helix to ß-hairpin/loop (Fig. 1f and g).

### ADME/PK properties of PAD4 inhibitor JBI-589

JBI-589 has excellent ADME properties and is orally bioavailable in mice. Intravenous (IV) and oral (PO) pharmacokinetics studies in mice revealed that the half-life was 8.0 h and 6.3 h, respectively (Fig. 1e).

Metabolic stability data suggested that JBI-589 has low-to-moderate turnaround under in vitro conditions using liver microsomes. Our results indicate that JBI-589 has weak or no propensity to inhibit the major Cytochrome P450s (CYPs) tested and predicted IC_50_ values are greater than 10 μM (Supplementary Table 2). Further, this compound exhibits an unbound fraction in the range of 5–10% in plasma across species. The compound exhibits low to moderate plasma clearance, as predicted by in vitro studies and high apparent volume of distribution (≥ total body water content) across species, which indicates that JBI-589 is highly distributed (Supplementary Table 3).

### Effect of JBI-589 on NET formation

**Figure 2:**
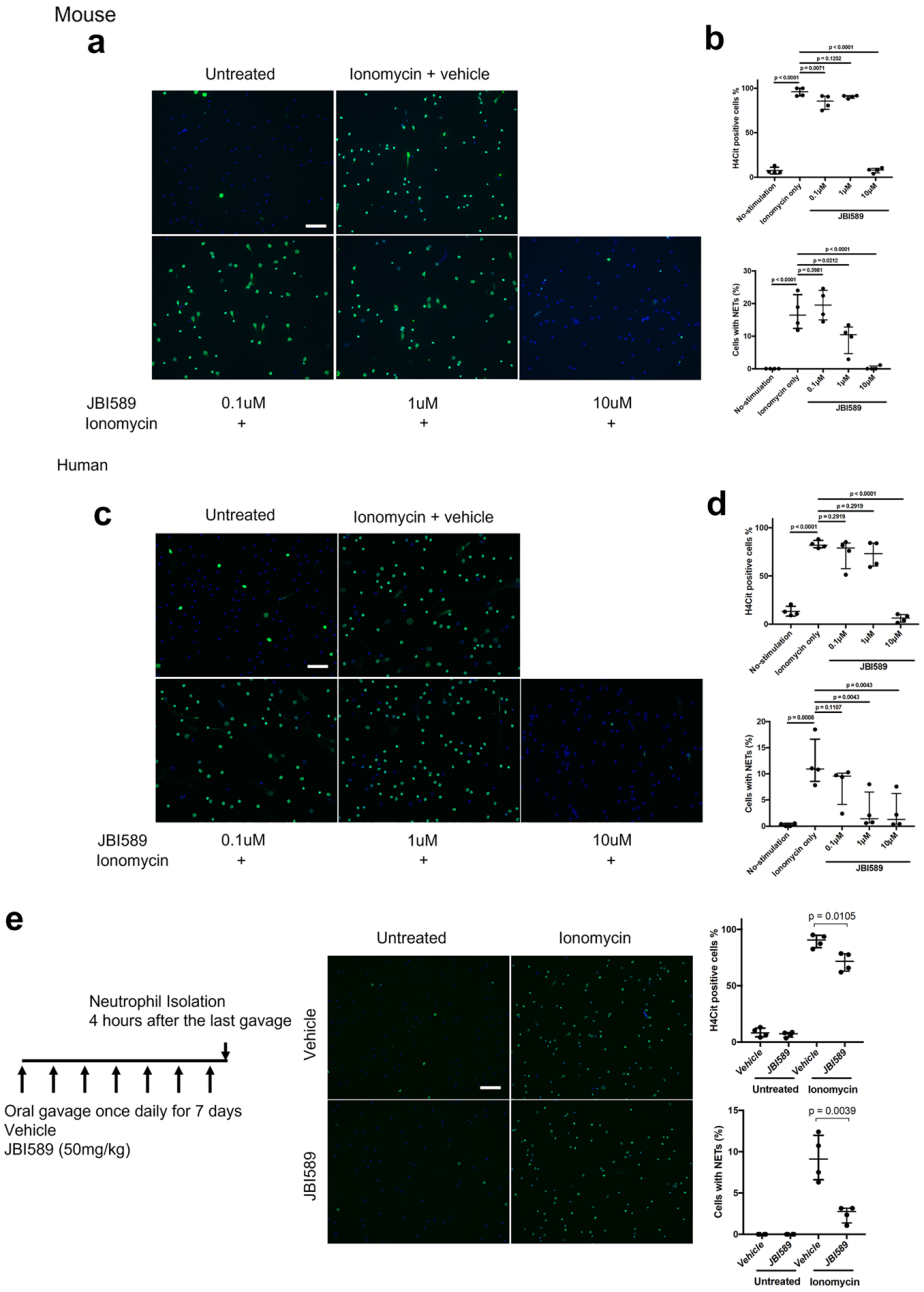
Inhibitory effects on citrullinated histone H4 and NET formation in vitro. (**a**) Mouse neutrophils with NET formation stained with citrullinated histone H4 (H4Cit) by Immunofluorescent microscopy. Isolated neutrophils from mouse peripheral blood were incubated with JBI-589 (0, 0.1, 1 and 10  µM) for 20 min followed by addition of ionomycin (4 µM) for 4 h (blue: Hoechst 33,342 (DNA), green: H4Cit antibody stain, scale bar = 100 μm). Representative of n = 4 experiments. (**b**) Quantifications of H4Cit positive cells (upper panel) and cells with NET formation (lower panel) by counting at least 200 cells per sample. (**c**) Human neutrophils with NET formation stained with citrullinated histone H4 (H4Cit) and visualized by immunofluorescent microscopy. Isolated neutrophils from mouse peripheral blood were incubated with JBI-589 (0, 0.1, 1 and 10  µM) for 20 min followed by addition of ionomycin (4 µM) for 4 h (blue: Hoechst 33,342 (DNA), green: H4Cit antibody stain, scale bar = 100 μm). Representative of n = 4 experiments. (**d**) Quantifications of H4Cit positive cells (upper panel) and cells with NET formation (lower panel) by counting at least 200 cells per sample. (**e**) Vehicle (water) or 50 mg/kg of JBI-589 was orally administered once daily for seven consecutive days (Left panel). Four hours after the last administration, mouse neutrophils were isolated and then a NET formation assay was performed with and without ionomycin (4 µM) in vitro. Representative images of H4Cit positive cells and cells with NET formation of n = 4 experiments (Middle). Quantifications of H4Cit positive cells (upper panel) and cells with NET formation (lower panel) was done using ImageJ and at least 200 cells were counted for each condition. The percentage of H4Cit positive cells was 88% and 83%, for vehicle and JBI-589 (*p* = 0.01) treated groups, respectively.

To determine the effects of JBI-589 on NET formation, an in vitro NET assay was performed. In mouse neutrophils, ionomycin stimulation induced more than 90% of H4Cit-positivity and NET formation in approximately 15% of neutrophils. Pretreatment with JBI-589 at 10 μM for 15 min markedly diminished H4Cit positivity and NET formation (Fig. 2a and b). In addition, the same effect was confirmed on human neutrophils (Fig. 2c and d). To evaluate the in vivo effects, JBI-589 at 50 mg/kg or vehicle (water) was orally administered twice daily for seven consecutive days. Four hours after the last administration, mouse neutrophils were isolated, and then a NET formation assay was performed. JBI-589 reduced both H4Cit positivity and NET formation significantly compared with vehicle (Fig. 2e).

### In vivo efficacy of JBI-589 on mouse arthritis models

We sought to investigate the effect of JBI-589 in disease models. Because PAD4 contributes to the pathogenesis of RA, we used murine arthritis models that are at least partially PAD4-dependent and mimic human RA with synovial tissue NET formation and the production of anti-H3Cit antibody: (Granulocyte-colony stimulating factor (G-CSF)-modified Collagen-Induced Arthritis (CIA) model in C57/Bl6J mice and standard CIA model in DBA/1 mice)^15,41^.

**Figure 3:**
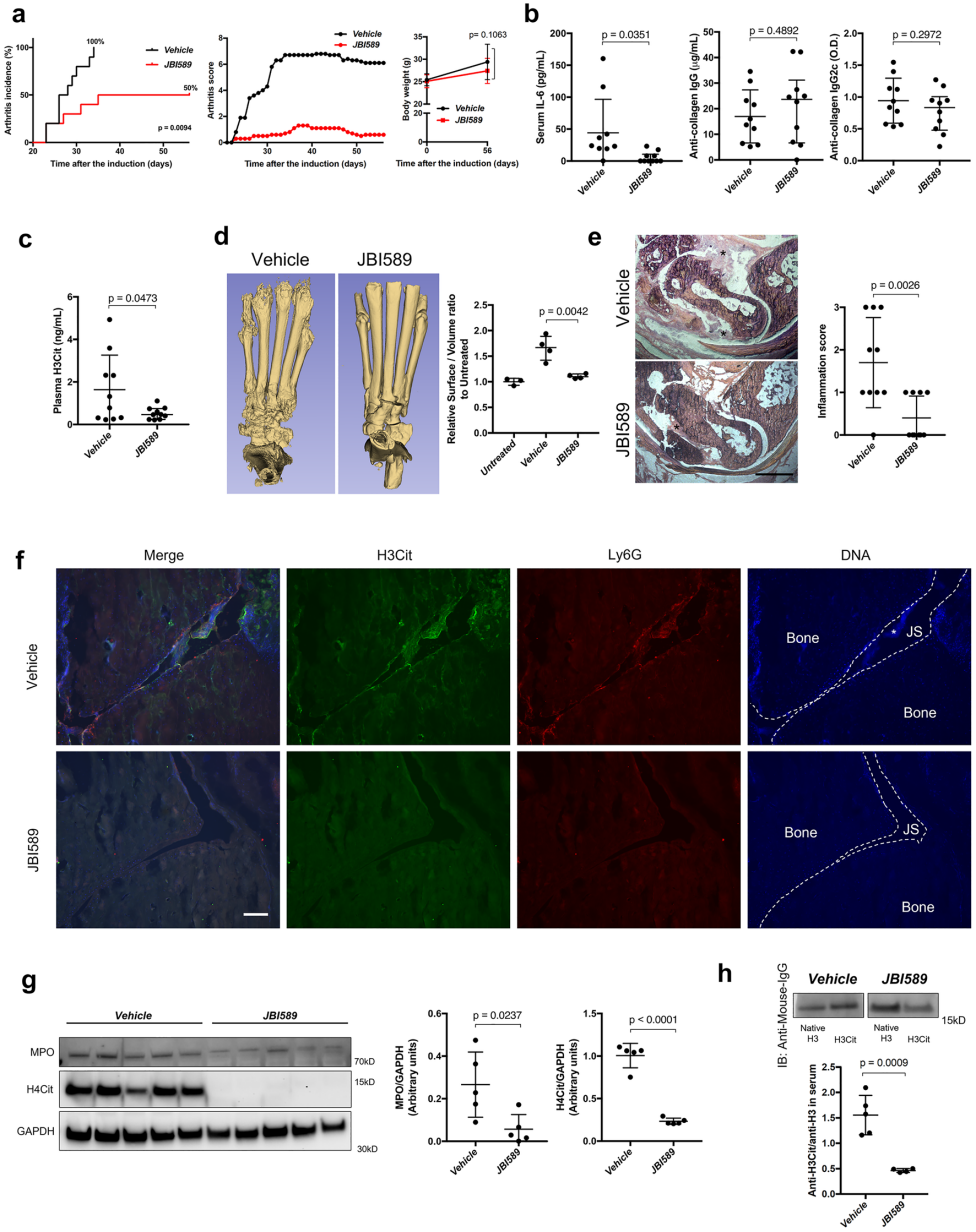
Efficacy of JBI-589 in the G-CSF-modified collagen-induce arthritis model in C57BL/6 mice. Vehicle (water) or 50 mg/kg of JBI-589 was orally administered twice daily from day 20 to day 56. (**a**) Arthritis incidence (left) and severity and body weight (right) of vehicle- and JBI-589-treated mice (n = 10:10). (**b**) Serum IL-6 and serum anti-collagen IgG and IgG2c antibody on day 56. (**c**) The changes in plasma levels of citrullinated histone H3 on day 22. (**d**) Representative images of micro-CT of ankle joints (left) and quantification of eroded surface of joints (right). (**e**) Representative images of H&E staining (left) and determination of inflammation in H&E stains by scale of 0 (no inflammation) to 3 (severe inflamed joint) depending on the number of inflammatory cells in the synovial cavity (exudate) and synovial tissue (infiltrate) (right). (*: Proliferated synovial tissue. scale bar = 500 μm) (**f**) Representative images of Ly6G and H3Cit immunostaining of ankle joints. (JS: Joint space, *: Synovial tissue, scale bar = 100 μm.). Images were obtained using a Zeiss Axiovert 200 M wide-field fluorescence microscope (Zeiss, Oberkochen, Germany) with the Zeiss AxioVision software (Version 4.6.3.0). (**g**) Western blot of MPO, H4Cit, and GAPDH (left) levels in synovial tissue (n = 5:5) was detected by ChemiDoc MP Imaging System (Bio-Rad Laboratories) and quantification by densitometry (middle and right, relative expression to GAPDH). (**h**) Representative images of blots with serum anti-native H3 antibody and anti-H3Cit antibody (upper) and quantification by densitometry (lower, anti-H3Cit/anti-native H3). Images were processed with Fiji/ImageJ software^61^ (National Institutes of Health) for the western blot and the microscopy image analysis.

Oral administration of JBI-589 twice daily from day 20 to day 56 showed a significant effect on arthritis incidence and severity (Fig. 3a) in the G-CSF modified CIA model. JBI-589-treated mice had reduced serum Interleukin -6 (IL-6) but comparative anti-Collagen Type II (CII) IgG and IgG2c antibody levels (Fig. 3b). JBI-589-treated mice showed lower plasma levels of H3Cit compared with vehicle-treated mice on day 22 (Fig. 3c). When evaluated by micro-computed tomography (micro-CT), we observed fewer areas of eroded surface of bones in JBI-589-treated mice (Fig. 3d) as compared to control mice. H&E staining of ankle joint to examine inflammation (exudate and infiltrate) revealed JBI-589-treated mice had alleviated severity of arthritis compared with vehicle-treated mice (Fig. 3e). Immunofluorescence microscopy revealed lesions positive for H3Cit and Ly6G and DNA within the joint space and on the bone surface in vehicle-treated mice but not in JBI-589-treated mice (Fig. 3f). Synovial tissue from JBI-589-treated mice contained less Myeloperoxidase (MPO) and H4Cit compared with that from vehicle-treated mice (Fig. 3g). JBI-589-treated mice also developed less serum anti-H3Cit antibody compared with vehicle-treated mice (Fig. 3h).

**Figure 4:**
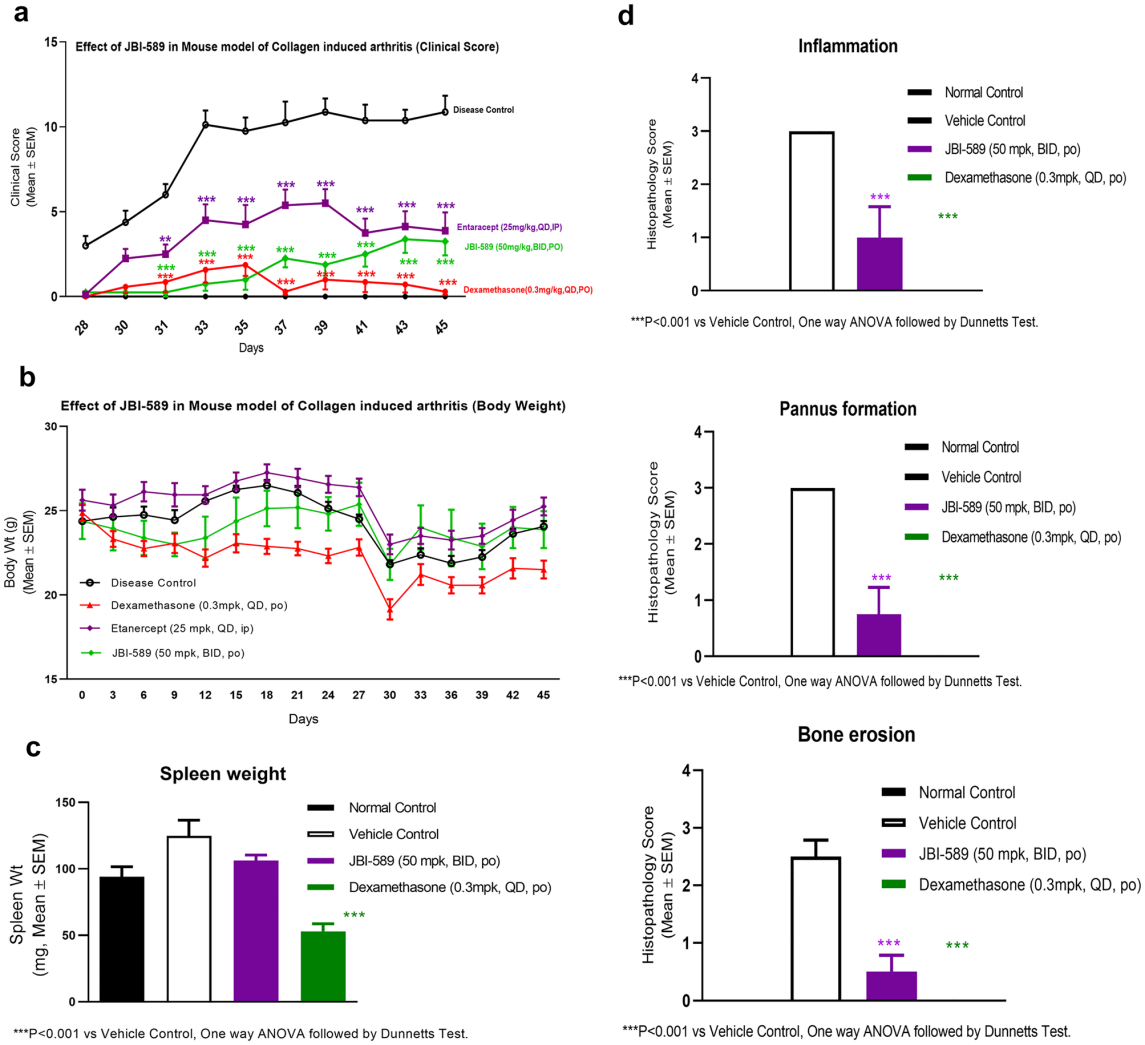
Effect of JBI-589 in the collagen-induce arthritis model in DBA/1 mice. Mice were dosed with either vehicle control (0.5% methyl cellulose + 0.05% tween 80) or JBI-589 at 50 mg/kg, PO twice daily from day 0 until day 45. Etanercept was administered at 25 mg/kg, IP from day 31–45. (**a**) Clinical score of mice from day 28 to day 45 (**b**). Body weight of mice from day 0 to day 45 (**c**). Effect of various treatments on spleen weight of mice on day 45 d. Histological scores determined by H&E staining in the paws of arthritic mice measuring inflammation, pannus formation and bone erosion.

To confirm the effects of JBI-589 on arthritis in another mouse arthritis model, we used a conventional CIA model using DBA/1 mice that are susceptible to arthritis development. Mice treated with vehicle control showed disease progression as expected where disease induction was observed from day 28. The maximum clinical score was reached on day 33 and was sustained until the end of the study (day 45). Prophylactic treatment with JBI-589 at the dose of 50 mg/kg (po, BID) for 45 days significantly decreased the clinical score, thus attenuating the development of arthritic symptoms (Fig. 4a). JBI-589 treatment had no adverse effect on body weight or spleen weight at end of study (Fig. 4b and c). Histopathological analysis revealed that oral administration of JBI-589 significantly decreased joint inflammation, pannus formation and bone erosion (Fig. 4d). Etanercept (Tumor necrosis factor inhibitor) showed a comparable inhibition of clinical score at the end of study (Fig. 4a), while showing no appreciable effect on inflammation, pannus formation or bone erosion (data not shown). Treatment with Dexamethasone (Steroidal anti-inflammatory drug) resulted in strong inhibition of clinical score which was associated with stronger reduction of body weight and spleen weight (Fig. 4b). Histopathological analysis revealed that treatment with Dexamethasone significantly decreased joint inflammation, pannus formation and bone erosion (data not shown).

## Discussion

These studies conducted by us highlights two significant advances in the field of PADs. First, we developed a highly PAD4-selective non-covalent inhibitor that is orally available. Second, the effectiveness of the inhibitor was confirmed by in vitro NET formation assay with human and mouse neutrophils cells and in vivo mouse arthritis models using two different kinds of mouse strain. In addition, the inhibition of NET formation, reduced NETs in synovial tissue and reduced anti-citrullinated histone H3 antibody in the G-CSF-modified CIA model provides additional evidence on the role of NETs in the pathogenesis of RA. Crystal structure of PAD4 bound with JBI-589 at 2.88 Å in calcium-free form, reveals binding mode same as reversible PAD4 inhibitor GSK199^18^ in partial calcium-bound form (4 calcium ions per monomer) and overlays within 0.53 Å RMS (root mean square) deviation. Key SAR (structure–activity relationship) observed for GSK199 was retained here as well. Primary amine of piperidine moiety preserves salt bridge against Asp-350, Asp-473 and interacts with His-471. JBI-589 is very selective for PAD4 and this can be corroborated with structure as the central part of the inhibitor is closely packed within 4 Å by Phe-634 which is not conserved in PAD 1–3, 6.

PADs have been thought to have specificity for their substrate although roles of various PAD isozymes in pathogenesis of different diseases is still emerging^42^. Therefore, the selectivity of the PAD inhibitor seems essential to focus on the appropriate target of the disease and to reduce the adverse off target effects. Previous studies using genetic methods and mouse disease models suggest that the role of PAD4 could be more important in RA than other PADs^43^ and our results showing that PAD4 inhibitor is sufficient to significantly reduce the incidence and severity of arthritis, are consistent with these previous findings.

Except for those drugs that target Janus kinase, the majority of the targeted disease-modifying anti-rheumatic medicines (DMARDs) now treated for RA are only available as intravenous injections^44^. Pan-PAD inhibitors and PAD4 inhibitors reported earlier also show poor oral bioavailability^1^. An orally available RA drug is essential to reduce the physical and psychological burden that patients experience with infusion therapy during the chronic ailments. One of the advantages of our inhibitor is that it is orally available across pre-clinical species, making it considerably simpler to administer for immune-related illnesses that are needed for chronic treatment.

Another major advantage of targeting PAD4 pathway is that, inhibition of this pathway does not lead to immune suppression. Gene knock out studies have clearly demonstrated that PAD4-deficient mice remain normal as compared to their PAD4 wild type counterparts with no increase in infection and no signs of immune modulation^45^. Therefore, unlike most of the current therapies that cause immunesuppression, PAD4 inhibitor is significantly better tolerated and probably more effective when it comes to combination therapies.

Anti-citrullinated protein antibodies (ACPA) are highly specific antibodies detected in RA that have been associated with a more aggressive disease course. Therefore, ACPA are used not only as a diagnostic marker but also as a prognostic marker^46^. Additionally, ACPA can promote osteoclast maturation and activation, leading to articular damages, including bone erosion^47^. Anti-H3Cit antibodies in sera of RA patients contribute significantly to ACPA^48^. PAD4 citrullinates multiple proteins, the likely source of citrullinated antigens that subsequently lead to the formation of ACPA as well as NETs which play a key role in initiating inflammatory changes^49^. Therefore, inhibition of PAD4 would lead to a decrease in ACPA and NET formation thereby minimizing subsequent articular damage. Accordingly, our results demonstrate decreased levels of NETs in synovial tissue and reduced serum anti-H3Cit antibodies in inhibitor-treated mice, clearly demonstrating that PAD4 inhibitors could be useful to impede pathogenesis in RA. We also hypothesize that PAD4 inhibitors and other synthetic and biological disease-modifying antirheumatic drugs may complement each other by lowering both systemic and local inflammation and ACPA antigens.

Other than RA, a role for PAD4 in many pathological conditions is emerging. Studies using Pad4 deficient mice demonstrated a benefit in deep vein thrombosis^50^, organ fibrosis^51^, wound healing^52^, skin transplantation^53^, endotoxemic shock^54^, pregnancy loss^55^, atherosclerosis^56^, kidney injury^57^, and vasculitis^58^. Also, cardiovascular diseases^59^ and cancer metastasis^60^, appears to be NET dependent. These diseases and conditions may well be explored with a selective PAD4 inhibitor such as JBI-589.

In conclusion, we identified a novel, orally available PAD4-selective inhibitor JBI-589, which prevented the formation of NETs and PAD4 dependent citrullination. Oral administration of the inhibitor reduced arthritis incidence and severity in mouse models including the generation of one of the ACPAs. PAD4 inhibitors might be potential therapeutic interventions against diseases associated with NETs and PAD4. Oral availability and lack of immune suppression makes this inhibitor suitable for treatment of chronic ailments. Scope for PAD4 targeted therapies appears huge and they could be the next generation therapeutics in the field of autoimmune disorders and inflammation.

## Methods

### Synthetic scheme preparation of JBI-589

Commercially available 1H-indole-2-carboxylic acid (1) was converted into weinreb amide (2) by coupling reaction with N,O-Dimethylhydroxylamine.HCl. The intermediate 2 was then converted into intermediate 3 by Grignard reaction using ethyl magnesium bromide. Bromination of intermediate 3 with phenyl trimethyl ammonium tribromide afforded bromo intermediate 4, which on base mediated cyclization with methyl 2-aminoisonicotinate gave cyclized intermediate 5. Indole N-alkylation of intermediate 5 with 4-fluoro benzyl bromide gave intermediate 6, which on hydrolysis with sodium hydroxide gave corresponding acid 7. T_3_P mediated coupling between acid 7 and tert-butyl (R)-piperidin-3-yl-l2-azanecarboxylate afforded amide 8, which on reduction with zinc powder and ammonium hydroxide gave the de-brominated product 9. Boc de-protection of intermediate 9 was carried out using TFA to afford the desired product JBI-589. The detailed synthetic protocol is given in supporting information (Supplementary Notes).

### Protein expression and purification

The full length of human PAD4 (hPAD4) Single nucleotide polymorphism (SNP)^24^ protein amino acids 1-663 was cloned into pET28a expression vector between NdeI and XhoI restriction sites with hexa histidine tag at N-terminus. The hPAD4 protein was over-expressed in *E. coli* (BL21 Chaperon assisted pG-Tf2 from Takara) system by using with 2YT medium; the cells were grown at 37 °C until the OD_600_ reached 0.6, then 1 mM Isopropyl ß-D-1 thiogalactopyranoside (IPTG) was added and the incubation temperature was lowered to 17.85 °C. After 24 h of induction, the cells were harvested with 6000 g for 10 min and then pellet was resuspended with the lysis buffer containing 100 mM Tris pH 7.2, 500 mM NaCl, 10 mM Imidazole, 5% (v/v) Glycerol. In this resuspended pellet solution, 0.1% lysozyme and 0.1% protease inhibitor were added and then it was applied into the homogenizer (GEA Niro Soavi Panda Plus 2000 Homogenizer). Homogenised solution was then centrifuged with 39,000 g at 4 °C for 1 h. The supernatant was filtered and loaded onto the pre-equilibrated Ni–NTA affinity column (Ni Sepharose 6 Fast Flow resin from Cytiva) using the resuspension buffer as described above; for initial washing 40 mM imidazole concentration was applied and for the elution 500 mM imidazole concentration was used. The eluted protein was concentrated and dialyzed overnight against the buffer 20 mM NaH_2_PO_4_/Na_2_HPO_4_ pH 7.5, 0.5 mM EDTA, 5% (v/v) Glycerol, 2 mM β-mercaptoethanol. After dialysis, the protein was loaded onto pre-equilibrated Q-Sepharose column (Q Sepharose Fast Flow resin form Cytiva), then the protein was eluted with 20 mM NaH_2_PO_4_ / Na_2_HPO_4_ pH 7.5 0.5 mM EDTA, 5% (v/v) Glycerol, 2 mM β- mercaptoethanol, 1 M NaCl. This eluted protein was concentrated; incubated with 10 mM CaCl_2_ for 30 min and then further purified by size-exclusion chromatography (HiLoad 16/600 Superdex 75 prep grade) with the buffer containing 10 mM Tris pH 8.5, 500 mM NaCl, 1 mM TCEP. Final purified protein was concentrated to 12.83 mg/ml. The protein quality was checked at every step by SDS-PAGE and the concentration was determined by using Nanodrop spectrophotometer.

### Thermal shift assay

The melting temperature (Tm) of hPAD4 protein was determined by fluorescence based thermal shift assay^25^. Tm is the temperature at which there would be equal concentrations of unfolded and folded proteins^26^. Experiment reaction mixture containing 1 µM hPAD4 protein, 5X SYPRO Orange Protein Gel Stain (Thermo Fisher Scientific Catalog No: S6650), JBI-589 compound at four concentrations 1 µM, 2 µM, 5 µM and 10 µM with final volume made up with protein storage buffers were mixed carefully. All experiments were done in triplicates at reaction volume of 50 μL. 1 µM protein concentration used was based on the results optimized from separate concentration scout experiments. The samples were heated from 15 to 95 °C with an increment of 1 °C per minute using CFX96TM Real-Time PCR Detection system—C1000 Thermal Cycler (Bio-Rad). Instrument filters were custom configured to the optimal emission (610 nm) and excitation (492 nm) wavelengths for SYPRO orange dye^25^. Increase in fluorescence signal emitted by SYPRO Orange was used as key indicator for monitoring protein denaturation upon gradual heating. The SYPRO Orange binds to exposed hydrophobic regions of protein undergoing thermal denaturation and corresponding fluorescence emissions are recorded as melt curves. The temperature corresponding to the inflection point, Tm was determined by calculating the first derivative from Melt curve using CFX manager 3.1 software (Bio-Rad). JBI-589 compound recorded Tm shift upto 7 °C at 1:5 protein-compound molar ratio.

### Crystallization and data collection

hPAD4 protein was screened in sitting drop vapour diffusion method (96-well, Intelli plate, Hampton research, USA) at 1:1 ratio (Protein: Reservoir) with various commercial crystallization screens at 6 mg/mL protein concentration. hPAD4 protein was incubated with JBI-589 compound at 1:4 protein-compound molar ratio for 1 h at 4 °C, prior to crystallization drop set. Small, plate-like crystals observed in 0.1 M HEPES pH 7.0, 5% (v/v) Tascimate pH 7.0, 20% PEG 4000 condition. Crystals matured in around three days at 22 °C. These crystals were cryo protected in reservoir solution with the addition of 16% (v/v) glycerol and flash frozen in liquid nitrogen. X-ray diffraction data was collected at PX1 beam lines of Soleil Synchrotron, France.

### Data processing and structure determination

The data was processed using XDS in I 1 2 1 space group (2.88 Å). The data was reduced with AIMLESS program of CCP4 suite (version 7.1.018)^27^. The structure was solved by molecular replacement with MOLREP (version 11.7.03) by using 4X8G PDB as start model, where one molecule of PAD4 was observed in asymmetric unit. Further model correction and electron density map inspection was carried out by COOT (version 0.9.8.1)^28^ and refinement by REFMAC5^29^ program. JBI-589 compound was fit into the density by using AFITT^30^ program (version 2.6.0.0). Final R-factors converged to Rwork(%)/Rfree(%), 20.5 /27.3. Confirmation of good stereochemistry was done by calculating Ramachandran plot using PROCHECK^31^. The atomic coordinates and structure factors for human hPAD4-JBI-589 have been deposited in the Protein Data Bank with the accession numbers 8GOD (Supplementary Table 1). All crystal structure figures were generated using PyMOL^32^.

### PAD4 biochemical assay

This assay relies on the ammonia produced when peptidylarginine deiminase 4 (PAD4) deiminates Nα-Benzoyl-L-arginine ethyl ester (BAEE) (Sigma, Catalog No: B4500), a non-natural substrate. The ammonia reacts with a detector i.e., ortho-phthaladehyde (OPA) (Sigma, Catalog No: P0657) resulting in a fluorescent product that is then analyzed at an excitation wavelength of 410 nm and an emission wavelength of 470 nm^33^. Full-length recombinant human PAD4 used in the assay was produced at Jubilant Biosys Ltd, Bangalore. PAD4 enzyme (125 nM) was diluted in Assay Buffer (100 mM HEPES, 50 mM NaCl, 2 mM DTT, 0.6 mg/ml BSA, pH7) and incubated with different concentrations of JBI-589 for 60 min at room temperature and then the reaction was initiated by the addition of substrate (1.5 mM BAEE (sigma) in 100 mM HEPES, 50 mM NaCl, 500 μM CaCl2, 2 mM DTT, pH7) then incubated with 1.5 mM BAEE substrate (Sigma) for another 60 min. The reaction was then stopped by the addition of detection buffer containing 50 mM EDTA, 2.6 mM ortho-phthalaldehyde and 2 mM DTT and fluorescence was measured. IC_50_ values were determined by sigmoidal dose–response curve (variable slope) using GraphPad Prism 5 software. For evaluating the activity of JBI-589 against PAD1, PAD2, PAD3 and PAD6 similar assay procedures were employed, with the exception that the enzymes were used at concentrations of 100, 200, 250 and 1000 nM, respectively. PAD6 (Catalog No: T1065-3) was obtained from Signal Chem, while PAD1 (Catalog No: 10784), PAD2 (Catalog No: 10785), and PAD3 (Catalog No: 10786) enzymes were purchased from Cayman Chemicals.

### Human neutrophil isolation

All blood samples from healthy donors who participated in this study were obtained after informed consent in accordance with relevant guidelines and regulations given by the ACE Independent ethics committee for Jubilant Biosys Ltd and Office of Clinical Investigations at Boston Children’s Hospital. The ACE Independent ethics committee, Bangalore, approved the use of human whole blood/PBMC/Enriched Subset in Jubilant Biosys Ltd investigations, Drug Controller General of India (DCGI) Reg No. ECR/141/Indt/KA/2013. Briefly, 10 mL K2EDTA vacutainers were used to collect fresh blood from healthy donors (BD vacutainer, Catalog No: BD 367525). A total of 50 ml of blood was taken from a single, healthy human donor. Neutrophils were isolated from human blood using the EasySep Direct Human Neutrophil Isolation Kit, according to the manufacturer's instructions (Stem Cell Technology, Catalog No: 19666). For investigations conducted at Boston Children’s Hospital, experimental procedure was approved by the Office of Clinical Investigations at Boston Children’s Hospital (protocol number IRB-P00003283). Informed consent was provided by donors. Neutrophils were isolated from heparinized blood by Polymorphprep (Axis-Shield, Oslo, Norway) in accordance with the manufacturer’s instructions. The purity of neutrophils was more than 90% as confirmed by the Wright-Giemsa stain.

### Mouse neutrophil isolation

Mouse peripheral blood was collected by retro-orbital puncture in ethylenediaminetetraacetic acid (EDTA) anticoagulated buffer supplemented with 1% endotoxin-free BSA in sterile PBS. Neutrophils were isolated by Percoll gradient centrifugation as described previously^34^. The purity of neutrophils was more than 90% as confirmed by the Wright-Giemsa stain.

### Peripheral blood mononuclear cell (PBMC) isolation from human whole blood

In a K2EDTA vacutainer, 50 mL blood from a healthy female human donor was collected, diluted 1:1 with PBS, and gently layered on Histopaque-1077. The tube was centrifuged at 400 g for 30 min at room temperature in a swinging bucket rotor without brakes or acceleration. After centrifugation, the tubes were gently removed from the centrifuge to avoid disturbing the layers of RBC at the bottom, PBMC in the middle, and clear supernatant at the top. Plasma and platelet-rich upper layer was aspirated using a sterile pipette leaving the mononuclear cell layer (buffy coat) undisturbed at the interphase. This buffy coat layer was transferred with care to a fresh conical 50 mL tube, resuspended in PBS buffer and the tube was centrifuged for 10 min at 300 g at room temperature, and the resultant cell pellet was resuspended in complete RPMI medium. After repeating this step twice, the resultant isolated cells were used for the experiments.

### Citrullination assay in human neutrophils

Citrullinated H3 Enzyme linked immunosorbent Assay (ELISA) was performed as per manufacturer’s instructions (Cayman chemicals, Catalog No: 501620). Neutrophils obtained as mentioned above were counted and cell suspension was prepared in complete RPMI media. Briefly, 0.5 million cells were seeded in a 24 well plate (Corning) and treated with various concentrations of JBI-589 for 3 h. The neutrophils were then stimulated with 25 µM of Calcium ionophore (Sigma, Catalog No: C7522) for 30 min. After incubation, cells were lysed with lysis buffer plus protease inhibitor cocktail and used for estimation of citrullinated histone H3 by ELISA. IC_50_ values were determined by sigmoidal dose–response curve (variable slope) in Graph Pad Prism 5 software.

### Cell Titer Glo viability assay

The CellTiter-Glo luminescent cell viability assay was performed on neutrophils/peripheral blood mononuclear cells according to the manufacturer’s instructions (Promega, catalog number: G7570)^35^. Breifly, 0.25 million cells of Neutrophils /PBMC were seeded in a black 96 well clear bottom plate. Compound was added at desired concentrations in 1% DMSO. Cells were incubated for 24 h in CO_2_ incubator. Post incubation, media was completely removed from all the wells. 50 µL/well of CTG was added (PBS + CellTiter-Glo Reagent (Promega) in 1:1 ratio). Plate was incubated at room temperature for 20 min on an orbital shaker to induce cell lysis and then for 10 min without shaking to stabilize luminescent signal. Luminescence was recorded with Perkin Elmer envision 2103 multilabel reader.

### ADME studies

Microsomal stability: Liver microsomes (0.5 mg/mL) from different species were incubated with JBI-589 (1 µM) with or without NADPH (1 mM) at 37 °C for 30 min in potassium phosphate buffer (pH 7.4) and then reaction was stopped with quenching solution (0.5% Formic acid in internal standard (IS) containing ACN). Resulting samples were centrifuged and supernatant was taken for LC–MS/MS analysis. Percent metabolism was calculated as follows: % Metabolized: [1 − (test area ratio/control area ratio)] *100.

Plasma protein binding: 10 µM of JBI-589 was mixed with plasma from different species (in sodium phosphate buffer, pH 7.4) was added to the plasma side of the 96-well micro-equilibrium dialysis device and sodium phosphate buffer (pH 7.4) was added to the other half of the device. The device was incubated at 37 ± 1 °C for 14 h with constant shaking. After the incubation time, plasma and buffer were collected from either side, quenched (0.5% formic acid in internal standard (IS) containing ACN), centrifuged and supernatant was used for LC–MS/MS analysis. Percent of unbound compound was calculated as follows: Percent unbound: [peak area ratio in buffer side/dilution factor]/peak area ratio in plasma side × 100; Peak area ratio: compound peak area/internal standard peak area.

CYP inhibition: Human liver microsome (0.1–0.5 mg/mL for various CYPs) and appropriate probe substrates (2.5–20 µM) were incubated in potassium phosphate buffer with or without JBI-589 for 5 min at 37 °C. The reaction was initiated by addition of NADPH (1 mM), incubated at 37 °C for 10 min and quenched (0.5% Formic acid in internal standard (IS) containing ACN). Samples were then centrifuged and taken for LC–MS/MS analysis. Percent change in metabolite formation for each substrate under above conditions using formula: Percent inhibition = [1 − (test area ratio/control area ratio)] *100.

### Mice PK study

Institutional Animal Ethical Committee (IAEC) of Jubilant Biosys Ltd (IAEC/JDC/2019/188R (for Mice) nominated by CPCSEA (Committee for the Purpose of Control and Supervision of Experiments on Animals) approved the mice pharmacokinetic experiments. Male Balb/c mice (~ 6–8 weeks old with body weight range of 22–25 g) were procured from Vivo Biotech, Hyderabad, India. Rodent feed (manufactured by Altromin Spezialfutter GmbH & Co. KG., ImSeelenkamp20, D-32791 Lage), and water was provided ad libitum. Intravenous (IV) and oral (PO) pharmacokinetics studies were done at doses of 2 mg/Kg and 10 mg/Kg, respectively at a dose volume of 10 mL/Kg. Sparse sampling was done with three mice at each time point. Blood (~ 100 µL) was collected from retro-orbital plexus at 0.12, 0.25, 0.5, 1, 2, 4, 8 and 24 h for IV and 0.25, 0.5, 1, 2, 4, 8, 10 and 24 h for PO route, in tubes containing K2 and EDTA as anticoagulant. It was centrifuged for 5 min at 10,000 rpm in a refrigerated centrifuge (Biofuge, Heraeus, Germany) maintained at 4 °C for plasma separation. For oral dosing of JBI-589 suspension formulation was prepared using 0.5% Tween-80 and 0.5% methyl cellulose (0.5% in water), while for IV dosing 5% DMSO, 5% Solutol:ethanol (1:1, v/v) and 90% normal saline was used.

### In vitro NET assay

Mouse or human neutrophils were resuspended in HEPES-supplemented phenol red-free RPMI1640 medium and 1.5 × 10^4^ cells were seeded per well in a 96-well plate. After incubating for 20 min at 37 °C and 5% CO2, the cells were stimulated with vehicle control or ionomycin (Sigma, Catalog No: I24222) (4 µM) for 4 h. Neutrophils were fixed with 4% ﻿paraformaldehyde (PFA), washed and permeabilized (0.1% Triton X-100, 0.1% sodium citrate) for 10 min at 4 °C. Subsequently, neutrophils were incubated with blocking buffer (2.5% BSA, 0.5% Tween-20 in 1 × PBS) at room temperature for an hour followed by overnight incubation with the primary antibody against citrullinated histone H4 (H4Cit, 1:250, catalog number: 07-596, Millipore, Burlington, MA). After washing 3 times with PBS, neutrophils were incubated with Hoechst 33342 (1:10,000, catalog number: H3570, Invitrogen). Images were obtained with a Zeiss Axiovert 200 M wide-field fluorescence microscope (Zeiss, Oberkochen, Germany) with the Zeiss AxioVision software. The percentages of H4Cit positive neutrophils and neutrophil extracellular traps (NETs) were calculated by quantifying more than 200 randomly selected cells. NET-positive cells were defined as cells with web-like chromatin structure and positive citrullinated histone H4 staining. The average percentage of cells was taken from duplicates in each sample.

### Animals

Wild type C57BL/6 mice were bred in-house. All mice were housed in the animal facility of Boston Children’s Hospital and were kept pathogen free. Experimental protocols were approved by the Institutional Animal Care and Use Committee of Boston Children's Hospital (Protocol number: 20-01-4096R). For the CIA study with DBA/1 mice, experiments were carried out at Jubilant Biosys Ltd, a AAALAC (Association for Assessment and Accreditation of Lab Animal Care International) accredited facility. Six- to eight-week-old DBA/1 male mice were purchased from The Jackson Laboratory (Bar Harbor, ME) and housed in Jubilant Biosys Ltd., animal facility under standard laboratory conditions with a 12-h light/dark cycle and access to food and water ad libitum. Prior to the study conduct, the Institutional Animal Care and Use Committee (IAEC/JDC/2021-242) reviewed and approved the procedures involving the care and use of animals.

### Granulocyte colony-stimulating factor (G-CSF)-modified collagen-induced arthritis model

C57BL/6 mice aged 8– 12 weeks were immunized with the emulsion of complete Freund's adjuvant (CFA, catalog number: 7023, Chondrex, Redmond, WA) and 100 μg of chicken type II collagen (catalog number: 20012, Chondrex) in a 1:1 mixture (total 100 μl) subcutaneously into the base of the tail on day 0^18^. The booster immunization of CII with incomplete Freund's adjuvant (catalog number: 7002, Chondrex) was performed on day 21. Recombinant human G-CSF (Filgrastim, Neupogen, Amgen, Inc., Thousand Oaks, CA) was injected at 10 μg peritoneally once daily from day 20 to day 23. Vehicle (water) or 50 mg/kg of JBI-589 was orally administered twice daily from day 20 to day 56. The severity of arthritis was evaluated using the following clinical scoring system for each limb: 0, normal; 1, swelling in one finger joint; 2, swelling in more than one finger joint or wrist or ankle joint; 3, swelling in the entire paw; and 4, deformity and or ankylosis. The maximum score was 16 per mouse. Two evaluators (one knew the group allocation, the other did not) independently scored arthritis and arrived at agreements on final scorings. At the end of the study, all mice were euthanized after giving anesthesia using 2% isoflurane (Patterson Veterinary, catalog number: 07–893-2374).

### Enzyme-linked immunosorbent assay (ELISA)

Anti-type II collagen IgG antibody and IgG2c antibody in serum were detected by ELISA^36^. Nunc MaxiSorp Flat-Bottom 96 well Plate (catalog number: 44–2404-21, Invitrogen, Carlsbad, CA) were coated with 10 μg/mL of chicken type II collagen (catalog number: 20012, Chondrex) in ELISA coating buffer (catalog number: 421701, BioLegend, Inc., San Diego, CA) overnight at 4 °C. After washing with PBS plus 0.05% Tween-20, the plate was blocked with 5% bovine serum albumin in PBS plus 0.05% Tween-20 for an hour at room temperature (RT). Serum was diluted at 1:50,000 in 0.5% BSA (in PBS with 0.05% Tween-20) and incubated on the plate for 2 h at RT. The plate was washed and incubated with goat anti-mouse IgG (H + L)-HRP conjugate diluted at 1:1,000 in 0.5% BSA in PBS-T (PBS with 0.05% Tween-20, catalog number: 1706516, Bio-Rad Laboratories, Hercules, CA) or goat anti-mouse IgG2c-HRP conjugate diluted at 1:1000 in 0.5% BSA in PBS-T (PBS with 0.05% Tween-20, catalog number: PA1-29288, Invitrogen), respectively, for 1 h at RT. After washing, 3,3',5,5'-tetramethylbenzidine (TMB) substrate solution (catalog number: N301, Thermo Fisher Scientific, Waltham, MA) revealed the anti-mouse IgG bound to chicken type II collagen. After stopping the reaction by adding sulfuric acid, the plate was read on a microplate reader at a wavelength of 450 nm. Mouse anti-chick type II collagen antibody (catalog number: 7005, Chondrex) was used as positive control and standard to quantify samples' concentration for the anti-type II collagen IgG antibody. Plasma levels of citrullinated-histone H3 were measured using the citrullinated Histone H3 (Clone 11D3) ELISA Kit (catalog number: 501620, Cayman, Ann Arbor, MI) in accordance with the manufacturer’s instructions. Serum levels of IL-6 were determined with ELISA MAX Deluxe Set Mouse IL-6 (catalog number: 431304, Biolegend) in accordance with the manufacturer’s instructions.

### Micro-computed tomography (Micro-CT) image acquisition and analyses

Scanning was performed in air using μCT 40 (Scanco Medical, Bassersdorf, Switzerland) at 80 kVp, 88 μA, 1000 ms integration time, and a voxel size of 6 μm. 3D reconstructions and analysis of images were performed with 3D Slicer^37^. The quotient of surface of bones (mm^2^) and volume of bones (mm^3^) of surface of ankle joints were calculated to evaluate the eroded surface.

### Hematoxylin and eosin (H&E) stain

H&E stain of joint tissue was performed following the previously described method^38^.

### Histological evaluation

Inflammation was scored on a scale of 0 (no inflammation) to 3 (severe inflamed joint) depending on the number of inflammatory cells in the synovial cavity (exudate) and synovial tissue (infiltrate)^39^.

### Immunofluorescence microscopy

Ankle joints harvested on day 56 were cryo-sectioned into 8 μm slices by the method of using a commercially available tape to stabilize joint tissue to enable sectioning of cartilage and bone tissues to be used for immunofluorescent staining without the need for demineralization^40^. Polyester Circuit Plating/Silicone Splicing Tape (JVCC, PPT-25C) and UV-curing liquid adhesive (Norland Optical Adhesive, 61 Norland Optical) were used as tape and adhesive to attach tape with the joint specimen to the glass slides. The Stratalinker UV crosslinker (Stratagene, La Jolla, CA, USA) crosslinked the adhesive and fixed tape on the glass slides. The sections were fixed in zinc fixative (100 mmol/L Tris–HCl, 37 mmol/L zinc chloride, 23 mmol/L zinc acetate, 3.2 mmol/L calcium acetate), permeabilized with 0.1% Triton-X and 0.1% sodium citrate for 10 min at 4 °C, blocked with 3% BSA in PBS-T at RT for an hour, and incubated with primary antibodies against H3Cit (1:1,000, catalog number: ab5103, Abcam, Cambridge, MA), or Ly6G (1:500, catalog number: 551459, BD Pharmingen, San Jose, CA), at 4 °C overnight. After washes, the sections were incubated with appropriate Alexa Fluor-conjugated secondary antibodies (1:1,500, catalog number: A-21206 (anti-Rabbit, Alexa Fluor 488) and A-21434 (anti-Rat, Alexa Fluor 555), Life Technologies, Carlsbad, CA) for 2 h at room temperature. Hoechst 33342 (1:10,000, catalog number: H3570, Invitrogen) was used to counterstain DNA. Images were obtained with a Zeiss Axiovert 200 M wide-field fluorescence microscope (Zeiss, Oberkochen, Germany) with the Zeiss AxioVision software (Version 4.6.3.0).

### Immunoblotting

Expressions of target proteins in synovial tissue on the joint surface of wrists and ankles were determined by immunoblotting. Synovial tissue was homogenized in radioimmunoprecipitation assay (RIPA) buffer (catalog number: 89901, Thermo Fisher Scientific) with protease inhibitor cocktails (catalog number: R0278, Sigma, St. Louis, MO) on ice. Following centrifugation at 20,000 g for 20 min at 4 °C, the protein content of the supernatant was determined by the Bradford protein assay (catalog number: 5000006, Bio-Rad Laboratories), and an equal amount of protein per sample was used. Protein lysate was resolved on gradient gels (Bolt 4–12% Bis–Tris Plus gels, catalog number: NW04122BOX, Life Technologies) and electroblotted on polyvinylidene difluoride (PVDF) membranes (catalog number: IB401001, Invitrogen) using the iBlot system (Thermo Fisher Scientific). After blocking with 5% BSA TBS-T buffer (0.05% Tween-20 in 1 × TBS) for 1 h at room temperature, the membranes were incubated with primary antibodies against myeloperoxidase (MPO, 1:500, catalog number: A0398, DAKO, Santa Clara, CA) or citrullinated histone H4 (H4Cit, 1: 1,000, catalog number: 07-596, Millipore, Burlington, MA) at 4 °C overnight, and subsequently with goat anti-rabbit IgG (H + L)- HRP (1:10,000, catalog number: 1706515, Bio-Rad) for 2 h at room temperature. Blots were developed with enhanced chemiluminescence substrate (catalog number: 32106, Thermo Fisher Scientific). Equal loading was confirmed by GAPDH (catalog number: G9545-200UL, 1: 10,000, Sigma). The intensity of the bands on blots was quantified using ImageJ software.

### Collagen induced arthritis model in DBA/1 mice

Induction of arthritis: Animals from study group, except the normal control (N = 8) received immunization. Primary immunization was done on day 0, followed by a secondary immunization on day 21, by intradermal injection at the base of tail. For primary immunization, bovine type II collagen (catalog number: 20021, Chondrex) (2 mg/ml) was dissolved in 0.05 M acetic acid. Complete freund’s adjuvant (CFA) was prepared by suspending M. tuberculosis (catalog number: 231141, Difco) (5 mg/ml) in incomplete freund’s adjuvant (catalog number: 263910, Difco) (IFA). Immunizing emulsion was prepared by emulsifying equal volume of Bovine type II collagen with CFA. For booster immunization, bovine type II collagen (2 mg/ml) was dissolved in 0.05 M acetic acid and emulsion was prepared by emulsifying equal volume of Bovine type II collagen with IFA^41^. On day 28 lipopolysaccharide (LPS—catalog number: L2630, Sigma; 50 μg/100 μl/ animal prepared in saline) was administered intraperitoneally to accelerate the onset of disease. Dexamethasone (Sigma, Catalog No: D1756) and Etanercept (Intas Pharmaceuticals, Batch No: 13030032, Injection formulation) were used in the study as standard of care.

Arthritic (Clinical) score: An arthritic (clinical) score was determined by grading severity of disease in each hind paw and forepaw according to a 0–4-point scale. Arthritic (clinical) scoring was based on the degree of peri-articular erythema and edema as well as deformity of the joints. The arthritic (clinical) score for each mouse was the sum of score of four paws with highest possible score being 16 for each mouse. Clinical score (from day 28) and body weights (from day 1) were measured on alternate days throughout the study. At the end of the study, all the mice were euthanized with CO_2_ overdose and hind paw was collected and fixed in 10% formal saline (for histopathology).

### Statistical analysis

Data were described with the mean and standard error for quantitative variables. We assessed the association between variables using unpaired t-test for quantitative variables. The cumulative incidence of arthritis was estimated with the Kaplan–Meier method and log-rank test. Holm-Sidak’s multiple comparisons test was used for more than two groups. All tests were two-sided or one-sided, and a *p*-value < 0.05 was considered significant. All statistical analyses were performed using GraphPad Prism ver. 7.0 or 9.0 (GraphPad Software, San Diego, CA, USA).

### Ethics statement

All the methods were carried out in accordance with the approved guidelines, and the full experimental procedures were carried out under the guidance of the Institutional Animal Care and Use Committee of Boston Children's Hospital and Jubilant Biosys Ltd. All methods are reported in accordance with ARRIVE guidelines.

## Supplementary Information

Supplementary Information.

## Data Availability

The datasets generated and analysed during this study are available from the corresponding author upon reasonable request. The atomic coordinates and structure factors data generated for human hPAD4-JBI-589 during the current study have been validated and deposited in the Protein Data Bank with the accession number [8GOD]. Use the following link to access the dataset: [https://www.rcsb.org/structure/unreleased/8GOD].
