# Supplementary material for: Alleviation of arthritis through prevention of neutrophil extracellular traps by an orally available inhibitor of protein arginine deiminase 4
Source: Sci Rep. 2023 Feb 23;13:3189. doi: 10.1038/s41598-023-30246-2 (PMC9950073; doi:10.1038/s41598-023-30246-2)
Supplement: Supplementary file 1 — Supplementary Information. [file 41598_2023_30246_MOESM1_ESM.docx]

**Alleviation of arthritis through prevention of neutrophil extracellular traps by an orally available inhibitor of protein arginine deiminase 4**

Chandru Gajendran^4^, Shoichi Fukui^1,2^, Naveen M Sadhu^4^, Mohammed Zainuddin^4^, Sridharan Rajagopal^4^, Ramachandraiah Gosu^4^, Sarah Gutch^1^, Saeko Fukui^1^, Casey E Sheehy^1^, Long Chu^1^, Santosh Vishwakarma^5^, Jeyaraj D.A^5^, Gurulingappa Hallur^5^, Denisa D Wagner^1,2,3^ and Dhanalakshmi Sivanandhan^4^*

^1^Program in Cellular and Molecular Medicine, Boston Children's Hospital, Boston, MA 02115, USA.

^2^Department of Pediatrics, Harvard Medical School, Boston, MA 02115, USA.

^3^Division of Hematology/Oncology, Boston Children's Hospital, Boston, MA 02125, USA.

^4^Jubilant Therapeutics Inc, Bedminster New Jersey, USA.

^5^Jubilant Biosys Limited, Bangalore, India

**Supplementary Note:**

The synthetic scheme and detailed synthetic protocol of JBI-589

Synthesis of (R)-(3-aminopiperidin-1-yl)(2-(1-(4-fluorobenzyl)-1H-indol-2-yl)-3-methylimidazo[1,2-a]pyridin-7-yl)methanone (JBI-589)

Step 1: Preparation of N-Methoxy-N-methyl-1H-indole-2-carboxamide (2)

To a stirred solution of 1H-indole-2-carboxylic acid (1, 25 g, 155.5 mmol) and N,O-dimethylhydroxylamine hydrochloride (30.26 g, 310.2 mmol) in dichloromethane (500 mL), triethylamine (107 mL, 775.6 mmol) and HOBt (36.7 g, 232.9 mmol) were added, followed by EDC.HCl (44.4 g, 232.6 mmol) at 0 °C. The reaction mixture was stirred at room temperature for 4 h. Water and dichloromethane were added to the reaction mixture. Dichloromethane layer was separated and washed with brine, dried over sodium sulphate and concentrated under reduced pressure. Diethyl ether (200 mL) was added to the resulting crude, stirred for 30 min and filtered to produce a white solid. This solid was dissolved in ethyl acetate, passed through celite bed and evaporated to give title compound as off white solid (2) (27 g, 87% Yield). MS (ESI): Mass calculated. for C_11_H_12_N_2_O_2_, 204.23; m/z found, 205.1 [M+H]^+^.

Step 2: Preparation of 1-(1H-Indol-2-yl)propan-1-one (3)

To a stirred solution of N-methoxy-N-methyl-1H-indole-2-carboxamide (2, 10 g, 49.01 mmol) in tetrahydrofuran (200 mL), 3M solution of ethyl magnesium bromide (49 mL, 147 mmol) was added drop wise at 0 °C. The reaction mixture was stirred at room temperature for 16 h. The reaction mixture was quenched with saturated ammonium chloride (100 mL) solution at 0 °C and extracted with ethyl acetate (500 mL). Organic layer was separated, washed with brine, dried over sodium sulphate and concentrated under reduced pressure. The crude was purified by flash column chromatography to give the title compound as off white solid (3) (4.5 g, 53% Yield). MS (ESI): Mass calculated. for C_11_H_11_NO, 173.22; m/z found, 174.1 [M+H]^+^.

Step 3: Preparation of 2-Bromo-1-(3-bromo-1H-indol-2-yl) propan-1-one (4)

To the stirred solution of 1-(1H-indol-2-yl) propan-1-one (3, 2.5 g, 14.45 mmol) in tetrahydrofuran (50 mL), phenyl trimethyl ammonium tribromide (16.3 g, 43.35 mmol) was added and stirred at reflux for 16 h. The reaction mixture was cooled to room temperature. Water (25 mL) was added and extracted with ethyl acetate (100 mL). Organic layer was washed with saturated sodium bicarbonate (20 mL) solution, brine (20 mL) and evaporated. Crude product was purified by column chromatography to give the title compound as brown solid (4) (4 g, 86% Yield). MS (ESI): Mass calculated for C_11_H_9_Br_2_NO, 331.01; m/z found, 333.9 [M+2H]^+^.

Step 4: Preparation of Methyl 2-(3-bromo-1H-indol-2-yl)-3-methylimidazo[1,2-a] pyridine-7-carboxylate (5)

To the stirred solution of 2-bromo-1-(3-bromo-1H-indol-2-yl)propan-1-one (4, 2 g, 5.24 mmol) and methyl 2-aminoisonicotinate (0.92 g, 5.24 mmol) in EtOH (20 mL), sodium bicarbonate (1.52 g, 26.2 mmol) was added and stirred at 90 °C for 16 h in a sealed tube. The reaction mixture was cooled to room temperature, water (50 mL) was added and extracted with ethyl acetate (2 X 50 mL). Combined organic extracts were washed with brine, dried over sodium sulfate and evaporated to give the crude product. The crude product was purified by flash column chromatography using 25-30% ethyl acetate in hexane to afford title product as brown solid (5) (0.45 g, 20.5% Yield). MS (ESI): Mass calculated for C_18_H_14_BrN_3_O_2_, 384.23; m/z found, 385.0 [M+H]^+^.

Step 5: Preparation of methyl 2-(3-bromo-1-(4-fluorobenzyl)-1H-indol-2-yl)-3-methylimidazo[1,2-a] pyridine-7-carboxylate (6)

To the stirred solution of methyl 2-(3-bromo-1H-indol-2-yl)-3-methylimidazo[1,2-a] pyridine-7-carboxylate (5, 0.2 g, 0.53 mmol) in N, N-dimethyl formamide (5 mL), cesium carbonate (0.51 g, 1.56 mmol) followed by 1-(bromomethyl)-4-fluorobenzene (0.15 g, 0.78 mmol) were added and stirred at 85 °C for 16 h. The reaction mixture was cooled to room temperature, water (20 mL) was added and extracted with ethyl acetate (50 mL). Combined organic extracts were washed with brine, dried over sodium sulfate and evaporated to give the crude titled product (6) (0.2 g, crude). MS (ESI): Mass calculated. for C_25_H_19_BrFN_3_O_2_, 492.35; m/z found, 492.2 [M+H]^+^.

Step 6: Preparation of 2-(3-bromo-1-(4-fluorobenzyl)-1H-indol-2-yl)-3-methylimidazo[1,2-a] pyridine-7-carboxylic acid (7)

To the stirred solution of methyl 2-(3-bromo-1-(4-fluorobenzyl)-1H-indol-2-yl)-3-methylimidazo[1,2-a]pyridine-7-carboxylate (6, 0.2 g, 0.4 mmol) in methanol (5 mL), 5N NaOH solution (1 mL, 2 mmol) was added and stirred at reflux for 1 h. The reaction mixture was cooled to room temperature, evaporated to dryness. Resulting crude was dissolved in water (10 mL), acidified using saturated citric acid solution and the compound was extracted with ethyl acetate (2 X 30 mL). Organic layer was separated, washed with brine (10 mL) solution, dried over anhydrous Na_2_SO_4_ and evaporated under vacuum to give the product as off white solid (7) (0.12 g, 62% Yield). MS (ESI): Mass calculated. for C_24_H_17_BrFN_3_O_2_, 478.32; m/z found, 476.0 (M-H) ^+^.

Step 7: Preparation of tert-butyl (R)-(1-(2-(3-bromo-1-(4-fluorobenzyl)-1H-indol-2-yl)-3-methylimidazo[1,2-a] pyridine-7-carbonyl) piperidin-3-yl) carbamate (8)

To a solution of tert-butyl (R)-piperidin-3-yl-l2-azanecarboxylate (0.075 g, 0.37 mmol) and 2-(3-bromo-1-(4-fluorobenzyl)-1H-indol-2-yl)-3-methylimidazo[1,2-a] pyridine-7-carboxylic acid (7, 0.12 g, 0.25 mmol) in dichloromethane (5 mL) at room temperature triethylamine (0.069 mL, 0.50 mmol) and propylphosphonic anhydride (T3P, 50% in ethyl acetate solvent) (0.18 mL, 0.37 mmol) were added. The reaction mixture was stirred at room temperature for 16 h. It was diluted with dichloromethane and washed with saturated sodium bicarbonate solution. Organic layer was separated, dried over sodium sulphate, and evaporated under reduced pressure to obtain crude. The crude product was purified by flash column chromatography using 5% methanol in dichloromethane to afford desired product (8) (0.11 g, 67% Yield). MS (ESI): Mass calculated. for C_34_H_35_BrFN_5_O_3_, 660.59; m/z found 660.2 [M+H]^+^.

Step 8: Preparation of tert-butyl (R)-(1-(2-(1-(4-fluorobenzyl)-1H-indol-2-yl)-3-methylimidazo[1,2-a]pyridine-7-carbonyl)piperidin-3-yl)carbamate (9)

To the stirred solution of tert-butyl (R)-(1-(2-(3-bromo-1-(4-fluorobenzyl)-1H-indol-2-yl)-3-methylimidazo[1,2-a]pyridine-7-carbonyl)piperidin-3-yl)carbamate (8, 0.11 g, 0.16 mmol) in methanol (6 mL), zinc powder (0.1 g, 1.60 mmol) was added followed by ammonium hydroxide (2 mL) and stirred at 90 °C for 16 h. The reaction mixture was cooled to room temperature, filtered through celite and the filtrate was evaporated. To the crude product, water (10 mL) was added and extracted with dichloromethane (30 mL). Organic layer was separated, dried over sodium sulfate and evaporated to give the crude product. The crude product was purified by flash column chromatography using 5% methanol in dichloromethane to afford the product as off white solid (9) (0.07 g, 72% Yield). MS (ESI): Mass calculated. for C_34_H_36_FN_5_O_3_, 581.69; m/z found, 582.3 (M+H)^+^

Step 9: Preparation of (R)-(3-aminopiperidin-1-yl)(2-(1-(4-fluorobenzyl)-1H-indol-2-yl)-3-methylimidazo[1,2-a]pyridin-7-yl)methanone (JBI-589)

To a stirred solution tert-butyl (R)-(1-(2-(1-(4-fluorobenzyl)-1H-indol-2-yl)-3-methylimidazo[1,2-a]pyridine-7-carbonyl)piperidin-3-yl)carbamate (9, 0.07 g, 0.12 mmol) in dichloromethane (5 mL) trifluoroacetic acid (0.5 mL) was added at 0 °C, then it was stirred at room temperature for 1 h. After completion of reaction, solvent was evaporated. The crude was dissolved in water (10 mL), neutralized with saturated sodium carbonate solution and extracted with dichloromethane. The combined organic layer was dried over anhydrous sodium sulfate, filtered and concentrated to give the crude product. The crude was purified by flash column chromatography using 8% methanol in dichloromethane to afford desired product (JBI-589) (0.012 g, 60 % yield) as off white solid.

^1^HNMR (400 MHz, DMSO-d_6_) δ (ppm): 8.40 (d, *J* = 7.2 Hz, 1H), 7.61-7.59 (m, 2H), 7.40 (d, *J* = 7.2 Hz, 1H), 7.12-7.08 (m, 1H), 7.02-6.98 (m, 6H), 6.75 (s, 1H), 5.86 (s, 2H), 4.05-4.01 (m, 1H), 3.57-3.51 (m, 1H), 2.97-2.94 (m, 1H), 2.78-2.71 (m, 1H), 2.62 (s, 3H), 2.02-1.98 (m, 2H), 1.84-1.81 (m, 1H), 1.67-1.61 (m, 1H), 1.44-1.41 (m, 1H), 1.25-1.21 (m, 2H). MS (ESI): Mass calculated. for C_29_H_28_FN_5_O, 481.58; m/z found, 482.2 (M+H)^+^. HPLC purity 99.68%.

**Supplementary Table S1 : X-ray data collection and refinement statistics**

| **Crystal Structure** | **Human PAD4 + JBI-589** |
| --- | --- |
| PDB id | 8GOD |
| Space group | I 2 |
| Unit Cell dimensions |  |
| *a*, *b*, *c* (Å) | 111.456, 60.879, 127.097 |
| α, β, γ (°) | 90.0, 104.58, 90.0 |
| **Data collection** |  |
| Source | Soleil Synchrotron, France  (PX1 Beamlines) |
| Wavelength (Å) | 0.97856 |
| Resolution range (Å) | 46.81 – 2.88 (3.04 – 2.88) |
| Observed Reflections | 131194 |
| Unique reflections | 18844 |
| Completeness (%) | 99.6 (99.4) |
| Multiplicity | 7.0 (7.2) |
| *<I>*/ σ <*I>* | 19.6 (2.8) |
| $\dagger$*R*_merge_ (%) | 5.1 (54.6) |
| ††*R*_pim_ (%) | 2.1 (21.8) |
| **Refinement** |  |
| Resolution range (Å) | 46.81 – 2.88 |
| No of reflections in |  |
| Working set | 17759 |
| Test set | 934 |
| Wilson B-factor | 101.75 |
| ‡*R*_work_ / §*R*_free_ | 0.205 (0.273) |
| No of atoms | 4469 |
| Macromolecules | 4429 |
| Ligand | 36 |
| Water | 4 |
| R.m.s deviations |  |
| Bond lengths (Å) | 0.0061 |
| Bond angles (°) | 1.4616 |
| Ramachandran analysis |  |
| Core (%) | 80.4 |
| Allowed (%) | 17.4 |
| Gen allowed (%) | 2.0 |
| Outliers (%) | 0.2 |
| **Average B-factors (Å^2^)** |  |
| Protein chains | 111.18 |
| Ligand | 119.175 |
| Water | 82.58 |

$\dagger Rmerge=\Sigma|I-<I>|/\Sigma I\times100,$ where *I* is intensity of a reflection and $<I>$ is its average intensity

$$\dagger\dagger Rpim=\Sigma\surd(\frac{1}{N-1})\times\Sigma|I-<I>|/\Sigma I\times100$$

$$\ddagger Rwork=\Sigma\left| \mathrm{Fo}-\mathrm{Fc} \right|/\Sigma|\mathrm{Fo}|\times100$$

§ Rfree is calculated on 5% randomly selected reflections, for cross-validation. Values in parentheses represent the highest resolution shell.

Supplementary Table S2. In vitro ADME parameters of JBI-589

| **Solubility (µM)** | 90 |
| --- | --- |
| **HLM/MLM/DLM/RLM % metabolized @ 30 min** | 25-50% |
| **PPB (% unbound)** | 5-10% |
| **CYP inhibition of CYP3A4, 2D6, 2C19, 1A2 and 2C9 (% inh @10 µM)** | <50 % |

**Supplementary Table S3. In vivo pharmacokinetic parameters of JBI-589 in mouse and rat**

| **Species** | **Route** | **Dose** | **t1/2** | **Cmax** | **C0** | **AUC0-t** | **AUC0-∞** | **CL** | **Vd** | **F** |
| --- | --- | --- | --- | --- | --- | --- | --- | --- | --- | --- |
|  |  | **mg/Kg** | **(h)** | **(ng/mL)** | **(ng/mL)** | **(ng∙h/mL)** | **(ng∙h/mL)** | **(mL/min/kg)** | **(L/kg)** | **%** |
| **Mice** | IV | 2 | 8 | 828 | 968 | 3957 | 4382 | 7.61 | 5.24 |  |
|  | PO |  | 6.3 | 874 | NA | 12474 | 13491 |  |  | 63 |
| **Rat** | IV | 6.32 | 419.3 | 451.3 | 1317 | 1394 | 24.3 | 13.2 |  |  |
|  | PO |  | 5.6 | 335 | NA | 3389 | 3579 | - |  | 51.4 |

**Supplementary Figure S1**


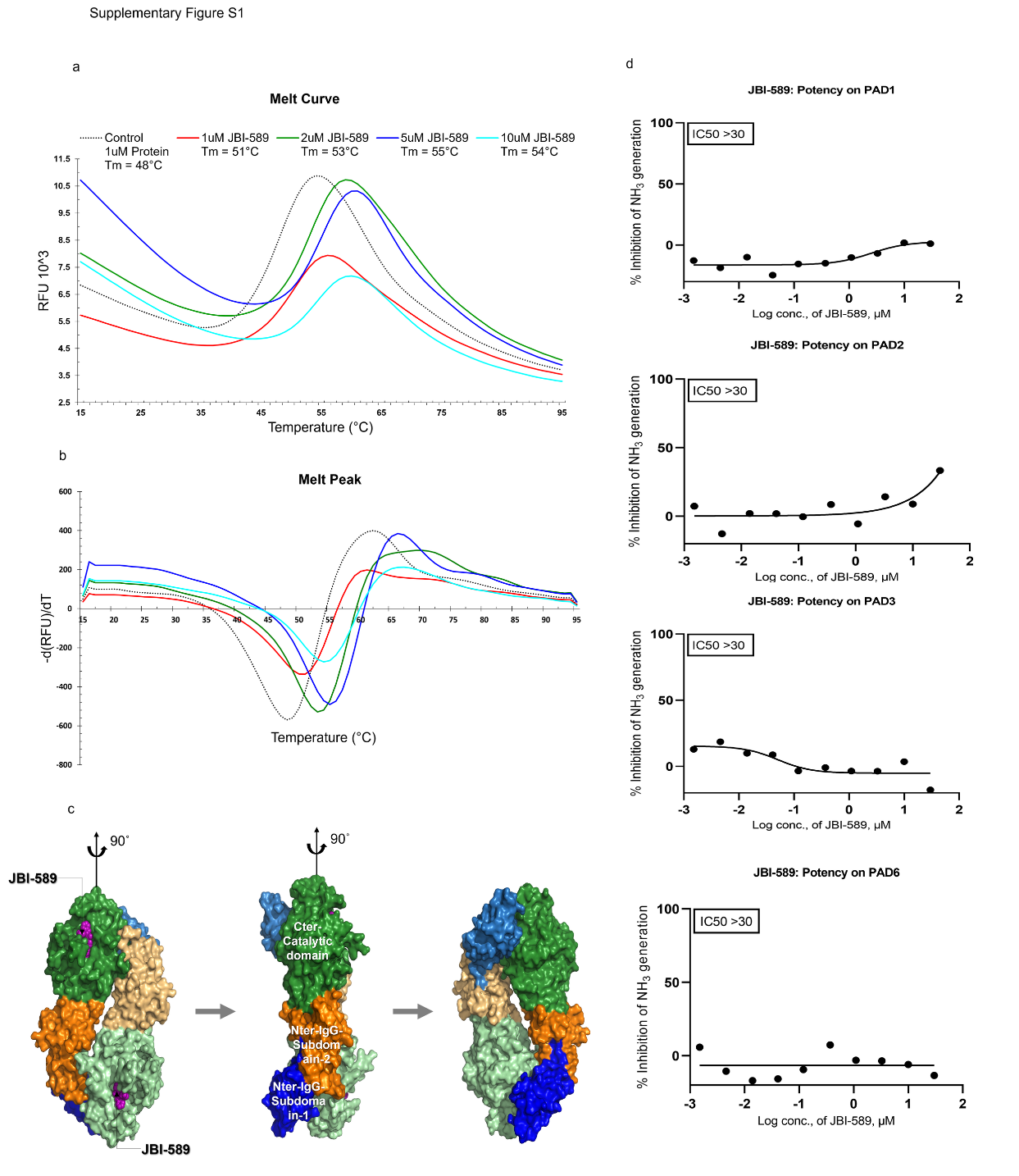


**Supplementary Figure S1:** Thermal shift assay at different protein: inhibitor stoichiometry. (a) Melt curve (b) Melt peak. Inflection points in melt curve and corresponding point in melt peak i.e., melting temperature (Tm) is right shifted in presence of increasing JBI-589 concentrations (thick line: red, green blue, cyan) when compared with apo PAD4 (dotted line: grey) and shows ~7˚C positive shift. (c) Co-crystal structure of human PAD4 homodimer with JBI-589 at 2.88Å resolution. Head-to-tail homodimer is shown in surface representation, in 3 orientations by rotating about 2-fold axis. N-terminal IgG subdomains 1,2 and C-terminal catalytic domains are colored differently. PAD4 inhibitor, JBI-589 shown in spheres. (d) Screening of JBI-589 in PAD1, PAD2, PAD3 and PAD6 selectivity biochemical assay measuring ammonia release.

**Supplementary Figure S2**


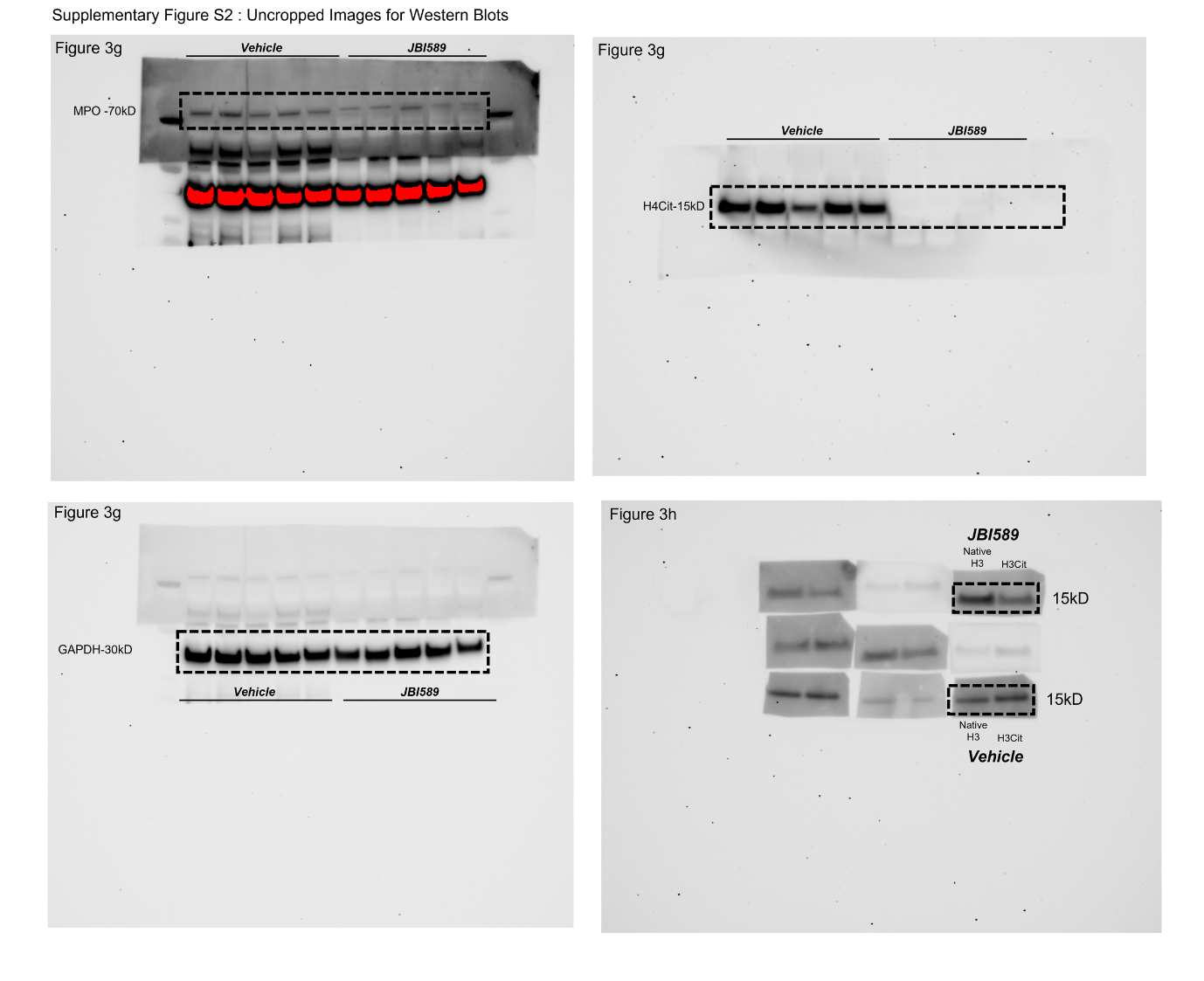


**Supplementary Figure S2:** Uncropped images of western Blots. (Figure 3g) Western blot of MPO (Top left), H4Cit (Top right), and GAPDH (bottom left). (Figure 3h) Upper from left: JBI589, Vehicle, JBI589; Middle from left: Vehicle, JBI589, Vehicle; Lower from left: Vehicle, JBI589, Vehicle. For each condition one representative image is shown in the manuscript.

Melt curve (b) Melt peak. Inflection points in melt curve and corresponding point in melt peak i.e., melting temperature (Tm) is right shifted in presence of increasing JBI-589
